# Fairer and healthier world for everyone – can lessons from the Dubrovnik Republic be applied to the COVID-19 pandemic?

**DOI:** 10.3325/cmj.2021.62.107

**Published:** 2021-04

**Authors:** Marina Lukežić, Ozren Polašek

**Affiliations:** University of Split School of Medicine, Split, Croatia *ozren.polasek@mefst.hr*

Quarantine, isolation, curfew, certificate… Although these words have become popular in 2020 and 2021, they are not new. If you had been around in the Republic of Ragusa, today's Dubrovnik and the surrounding districts in Croatia, in the 14th and 15th century you would have heard them being used on a daily basis.

In 1348, the city was severely affected by the plague, which might have killed as much as two thirds of the city population (1). In response to this and other epidemics, numerous counter-mechanisms have been developed. The Republic had a small population that it was determined to protect. The political ideals of liberty, prosperity, stability, longevity, and social tolerance strongly affected the goal of health care for all (2). One of the first European hospices for the poor (*Hospedal del comun*, *per beneficio dei poveri amalati*), was founded in 1347, while the earliest recorded Health Office was founded in 1390. The Republic was also among the first to employ and pay physicians, probably as early as 1280 (*medicus salariatus*); this meant that all citizens had free and fair access to health care and it effectively abolished the traditional payment to a physician – a chicken (3). The Republic was at the forefront of wholly understanding the inseparable link between population health and economy.

However, a true advance took place in 1377, when the first “*trentina*,*”* thirty days of isolation, was established. This would later become known as quarantine (4). In the face of new potential epidemics, the Republic could have closed the gates, stopped commerce, and tried to wait the epidemic out. However, for Dubrovnik, which lacked land and resources in its nearby surroundings, this was not an option. The city could not simply stop trading; that would have led to famine and financial ruin. Faced with such contradictory demands, a society that valued conservatism and continuity adopted a pragmatic solution: it embraced the plague control measures that permitted trading to continue, but on a smaller scale (2).

*“Veniens de locis pestiferis non intret Regusium vel districtum”* – “those arriving from plague-infected areas shall not enter Dubrovnik or its district” (4). The newcomers had to spend 30 days in isolation, initially on nearby islands, later in a separate building at the port entrance. This measure ensured that plague would not spread uncontested into the city. The Republic also introduced the role of *officiales contra venientes de locis pestiferis* and *officiales ad providendum super venientibus de locis pestiferis*, who were called *caxamorti*, *chazamorbi,* or later *cazamorti* (5). They were responsible for the epidemic response management, including assigning *trentina*, issuing certificates, collecting information about citizens, collecting traveler reports about plague outbreaks from other countries, as well as prohibiting free movement of the citizens during epidemics, unless the movement was dedicated to commerce (6).

Each new wave of plague led to the introduction of new measures, and each response seemed like a military operation, with a clear chain of command and society-wide response. Cazamorti were accompanied by armed guards, ensuring the generalized, war-like adherence to anti-epidemic measures (2). However, Dubrovnik never implemented the harsher anti-epidemic measures imposed in Milan, including walling in the affected families in their homes in order to restrict the plague spread (7). Instead, the anti-plague measures were firmly rooted in the social, environmental, economic, and political structures of the Republic; each new episode caused a substantial societal strain, but also gave rise for the measures that contributed to prosperity (2). The extent of social cohesion is clearly reflected in another domain, as the Republic issued pensions to physicians who remained in the city longer than expected; the first such case was recorded as early as 1399 (8).

These anti-epidemic measures shielded the Republic from two large waves of the plague (1575–77 and 1630), which ravaged modern-day Italy (9). For a while, it seemed that Dubrovnik had escaped the historic grip of plagues. However, not entirely. In 1526, the bustling city, usually noisy and overflowing with dockworkers loading and unloading goods from all sorts of ships – brigantines, carracks, grips, galleons, or caravels – as well as from overland caravans, suddenly fell eerily silent (2). Although this comment originally refers to the last recorded plague epidemic, the same happened nearly 500 years later, in 2020. This time, the silence was caused by the SARS-CoV-2 virus.

The Croatian national response to this modern-day plague largely relied on lockdown and systematic epidemic control measures. The initial wave of spring 2020 was countered by one of the strictest quarantines in the world, as estimated by the Oxford stringency index (10). The introduced measures were largely supported and accepted by the population (11). In the more recent waves, autumn 2020 and spring 2021, the Croatian Government imposed less stringent measures (with an average stringency index for August 2020-April 23, 2021 of 40.7, compared with 45.2 in the neighboring Bosnia and Herzegovina, 56.6 in Serbia, 62.8 in Hungary, and 68.4 in Slovenia) (10). Since the beginning of the pandemic until April 23, 2021, the Croatian infection rate was at 87% of the average of these neighboring countries, mortality rate at 84%, and the testing rate at 95% (12). However, here we have to take into consideration that the unified and harmonized excess mortality information in the EUROMOMO database is only available for Slovenia and Hungary (13), preventing direct comparisons. Although at this point we are unable to make generalized conclusions about the COVID-19 pandemic, one cannot help but wonder if this favorable result was, at least partly, the product of the long-lasting tradition of public health in Croatia. This tradition originated from the Dubrovnik Republic, extended across prominent figures like Andrija Štampar, who above all advocated equity as a prerequisite of attaining true population health (14), and was further influenced by the experiences of the 1991-1995 war.

Unfortunately, these benefits did not translate into SARS-CoV-2 vaccination coverage; on April 23, 2021, Serbia had 19% of the population fully vaccinated, Hungary 16.1%, Slovenia 8.4%, while Croatia had only 3.9% (15). This clearly shows that the epidemic and our response to it is a multidimensional problem, and taking a single or only a few epidemic-related indicators into consideration can easily lead to biased conclusions.

One thing is certain. We are still plagued by the lack of relevant and timely information. The data available in the public domain are insufficiently harmonized, making the direct comparisons biased, especially across countries and different timelines. On the other hand, our global response to the pandemic remains patchy and consequently less effective than it should be (16), especially in the domain of vaccine delivery and distribution (17). This lack of evidence-based, equitable, and global response to the pandemic puts all of us at an increased risk of new variants being generated and will surely extend the need for anti-epidemic measures. In turn, novel variants may increase the risk of more severe burden on health care (18), or even vaccine breakthrough, causing infections even in fully vaccinated individuals (19), and effectively increasing the risk of emerging, recurrent epidemic waves. Now more than ever, we must strive to achieve fairer and healthier world for everyone (20). Adhering to the tested and resolute approaches in epidemic control, such as the ones recorded in the Dubrovnik Republic, relying to evidence-based medicine methods, and achieving global vaccination outreach remain the best approach to timely ending this pandemic.
